# Medication Choices in Children With Tic Disorders in Mainland China, Macao, Hong Kong, and Taiwan: Perspectives of Guardians and Physicians

**DOI:** 10.3389/fphar.2022.852414

**Published:** 2022-05-03

**Authors:** Chunsong Yang, Yaya Yang, Lingli Zhang, Li Zhao

**Affiliations:** ^1^ Department of Pharmacy, Evidence-based Pharmacy Center, West China Second Hospital, Sichuan University, Chengdu, China; ^2^ Key Laboratory of Birth Defects and Related Diseases of Women and Children (Sichuan University), Ministry of Education, Chengdu, China; ^3^ Department of Health Policy and Management, West China School of Public Health/West China Fourth Hospital, Sichuan University, Chengdu, China

**Keywords:** pediatricians, aripiprazole, tiapride, doctors, dose

## Abstract

**Objective:** Survey pediatricians and guardians of children with tic disorder on medication needs and choices.

**Methods:** We designed a cross-sectional survey for pediatricians in mainland China, Hong Kong, Macao, and Taiwan, as well as for the guardians of patients with tic disorder from West China Second University Hospital. We collected and analyzed information on clinicians’ medical behavior and medication choices and on guardians’ knowledge of tic disorder, medical treatment behaviors, and medication choices and needs.

**Results:** We collected responses from 242 physicians and 610 guardians. For patients with tic disorder and without comorbidities, the first-line drugs selected by physicians were tiapride (60.74%), clonidine (32.64%), haloperidol (25.62%), aripiprazole (16.53%), and sulpiride (12.4%). Physicians reported making medication choices by considerations such as clinical guidelines, clinical efficacy, a low incidence of adverse drug reactions, sufficient clinical research evidence, convenient dosage forms, and patient adherence. Guardians reported making medication choices by considerations such as a low incidence of adverse drug reactions, physician recommendations, clinical efficacy, dose, dosage forms, and the convenience and steadiness of obtaining the medication. However, guardians exhibited insufficient knowledge of tic disorder and treatment options.

**Conclusions:** Physicians and patient guardians differ in their considerations when selecting medications, highlighting a gap in optimizing treatment.

## 1 Introduction

Tic disorder (TD) is a common childhood neuropsychiatric disorder characterized by motor or vocal twitching in one or more parts of the muscles and is sudden, involuntary, repeated, rapid, and purposeless (Yang et al., 2020). TD is categorized as transient, chronic, Tourette syndrome, or undefined (Liu et al., 2020).

The prevalence of transient TD, chronic TD, and Tourette syndrome in children has been estimated at 2.99, 1.61, and 0.77%, respectively, and appears to be more than four times higher in boys (1.06%) than in girls (0.25%) (Knight et al., 2012). In China, the prevalence of transient TD, chronic TD, and Tourette syndrome has been reported as 1.7, 1.2, and 0.3%, respectively (Yang et al., 2016).

TD patients often suffer from comorbidities that affect their physical and mental health. Approximately 30–50% of patients with TD are diagnosed with attention-deficit/hyperactivity disorder, and 10–50% of patients are estimated to have obsessive-compulsive disorder (Kurlan et al., 2002; Hirschtritt et al., 2015). Other comorbidities include sleep disorders, learning difficulties, anxiety, and depression. Patients with TD have an overall lower quality of life than children without TD (Conelea et al., 2011; Eddy et al., 2011; Evans et al., 2016).

Drug therapy is the main treatment to control the symptoms of TD in children, but medication choices vary by country and physician preferences (Waldon et al., 2013). A survey of 22 European experts (Roessner et al., 2011) recorded support for risperidone, clonidine, aripiprazole, and pimozide. A survey of Canadian physicians (Cothros et al., 2019) reported that aripiprazole, risperidone, and clonidine were the most commonly prescribed drugs for TD, but the use of risperidone was decreasing. A survey of 110 Chinese physicians (Lu et al., 2020) showed support for clonidine, aripiprazole, and tiapride as the preferred drugs for newly diagnosed TD cases with moderate chronic TD. Other surveys of drug choices for TD did not investigate factors related to medication choice and polled physicians but not patient guardians.

In addition, patient and guardian awareness of TD is important for controlling the condition, but research has rarely focused on guardian awareness of the disorder, medical treatment behaviors, medication choices, or patient needs. Therefore, we assessed these factors from the perspective of both guardians and physicians so as to improve guardian participation in treatment decision-making and the clinical outcomes.

## 2 Materials and Methods

### 2.1 Participants

Pediatricians from major hospitals in China who were members of child development and behavior groups of the Chinese Pediatric Society in Chinese Medical Association were included in the survey. Pediatricians were included if they were in active medical practice, without limitation of professional title or age, and if they prescribed medication for patients with TD. Interns, medical students, and trainees receiving standardized training were excluded.

Patients with TD from the outpatient department of pediatric neurology of West China Second University Hospital, Sichuan University, were included. Patients under 18 years of age who had been diagnosed with TD according to DSM-IV diagnostic criteria and whose guardians agreed to participate and sign the informed consent were included. Patients were excluded if they exhibited cerebral palsy, meningitis, motor language development lags, nail-biting, restless legs syndrome, myasthenia gravis, Brown syndrome, or other neuropsychiatric conditions.

### 2.2 Data Collection

Questionnaires for physicians collected data in three categories: basic information (sex, education level, professional title, years of medical service, and province), medical behavior (tic assessment methods, common treatment methods, and treatment goals), and prescribing behavior (preferred drugs and considerations in selecting drugs). Questionnaires for guardians collected data in three categories: basic information (patient age, disease duration, family history, type of tic, and comorbidities), guardian’s cognition of TD (understanding TD pathways, TD classification, symptoms and characteristics, pathogenic factors, common treatment methods, and treatment duration), and guardian’s medical behavior and medication choices (department of first visit, time to first treatment, treatment methods, and involvement in medication choices).

### 2.3 Data Analysis

Questionnaires with incomplete contents were excluded from the analysis. The mean (± standard deviation) or median was used to describe quantitative variables. The frequency or composition ratio was used for categorical variables. Tic assessment methods, treatment goals, and treatment strategies were assigned a numeric score of 1 (“very unimportant”), 2 (“not important”), 3 (“neutral”), 4 (“important”), or 5 (“very important”). Factors in medication choice were evaluated on the same scale. Data analyses were performed in SPSS version 22 (IBM SPSS, Armonk, NY, United States).

### 2.4 Ethical Considerations

The study protocol conformed to the Helsinki Declaration and was approved by the Office of Research Ethics Committees of West China Women’s and Children’s Hospital. All participants voluntarily took part in the study and provided informed consent.

## 3. Results

### 3.1 Survey of Physicians

#### 3.1.1 Physician Information

A total of 242 questionnaires were collected, and all contained complete information (effective rate: 100%). Participating physicians were from 24 provinces in eastern, central, and western China and from Hong Kong, Macao, and Taiwan. Almost three quarters (73.55%) were female, and almost all (97%) possessed at least one university degree. Sixty percent of participating physicians had professional titles of deputy senior or above, 69% had been practicing medicine for more than 10 years, and 75.21% worked at Grade III, Level A hospitals (Table 1).

**TABLE 1:** Demographic information of pediatricians (N = 242).

Content	Number (n)	Constituent ratio (%)
Sex		
Male	64	26.45
Female	178	73.55
Education background		
Bachelor’s degree	125	51.65
Master	85	35.12
PhD	32	13.22
Professional title		
Junior title	37	15.29
Intermediate title	59	24.38
Deputy senior title	77	31.82
Senior title	69	28.51
Time spent in clinical work		
1–5 years	34	14.05
6–10 years	41	16.94
11–20 years	75	30.99
≥21 years	92	38.02
Grade of affiliated hospital		
Grade III, Level A hospital	182	75.21
Grade III, Level B hospital	8	3.31
Grade II, Level A hospital	26	10.74
Grade II, Level B hospital	15	6.20
Others	11	4.55
Affiliated departments		
Pediatric neurology department	12	4.96
Child psychiatry department	10	4.13
Department of developmental behavioral	17	7.02
Child psychological counseling department	9	3.72
Department of children healthcare	62	25.62
Pediatric department	127	52.48
Others	5	2.07

#### 3.1.2 Medical Behavior

The most common methods for evaluating tics used by pediatricians were observation of tic symptoms (4.55 points) and reference to past medical history (4.39 points), followed by the tic comorbidities scale (3.96 points), functional examinations (3.91 points), and the tic specificity scale (3.89 points). Most common treatment goals were improving overall function (4.42 points), reducing tic frequency (4.39 points), alleviating comorbidities (4.30 points), and eliminating tics (3.98 points). The most commonly used treatment tactic reported was providing strategies to help patients manage tics (4.39 points), followed by oral or written education of parents (4.33 points), drug treatment (4.13 points), and surgery (2.16 points; Table 2).

**TABLE 2:** Evaluation methods, treatment goals, and treatment strategies of tic (N = 242).

Topic/Option	Very Unimportant n (%)	Unimportant	Neutral	Important	Very Important	Average
Evaluation Methods of Tic						
Observe tic symptoms	5 (2.07)	1 (0.41)	10 (4.13)	67 (27.69)	159 (65.7)	4.55
Reference to past medical history	5 (2.07)	1 (0.41)	19 (7.85)	86 (35.54)	131 (54.13)	4.39
Tic comorbidities scale	7 (2.89)	7 (2.89)	51 (21.07)	101 (41.74)	76 (31.40)	3.96
Various functional examinations	6 (2.48)	8 (3.31)	54 (22.31)	107 (44.21)	67 (27.69)	3.91
Tic specificity scale	9 (3.72)	13 (5.37)	50 (20.66)	94 (38.84)	76 (31.40)	3.89
**Treatment goals**						
Improve overall function	5 (2.07)	3 (1.24)	14 (5.79)	83 (34.30)	137 (56.61)	4.42
Reducing tic frequency	5 (2.07)	1 (0.41)	17 (7.02)	90 (37.19)	129 (53.31)	4.39
Alleviating comorbidities	4 (1.65)	2 (0.83)	24 (9.92)	100 (41.32)	112 (46.28)	4.30
Eliminate tic	9 (3.72)	6 (2.48)	56 (23.14)	80 (33.06)	91 (37.60)	3.98
**Treatment strategies**						
Provide strategies to help patients manage tics	5 (2.07)	2 (0.83)	21 (8.68)	80 (33.06)	134 (55.37)	4.39
Oral or written education of parents	6 (2.48)	3 (1.24)	26 (10.74)	78 (32.23)	129 (53.31)	4.33
Drug treatment	7 (2.89)	3 (1.24)	35 (14.46)	103 (42.56)	94 (38.84)	4.13
Surgery	111 (45.87)	39 (16.12)	55 (22.73)	17 (7.02)	20 (8.26)	2.16

Preferred treatment methods for patients without comorbidities were psycho-behavioral therapy (86.36%, 209/242), educational interventions (73.97%, 179/242), and drug therapy (68.18%, 165/242). For children with TD and comorbidities, the commonly used treatment methods were drug therapy (89.26%, 216/242), psycho-behavioral therapy (85.12%, 206/242), and educational interventions (71.49%, 173/242).

#### 3.1.3 Preferred Drugs and Influencing Factors

For patients without comorbidities, the first-line drugs were tiapride (60.74%), clonidine (32.64%), haloperidol (25.62%), aripiprazole (16.53%), and sulpiride (12.40%). For patients with TD and attention-deficit/hyperactivity disorder, the preferred drugs were tiapride (50.83%), clonidine (32.64%), haloperidol (25.21%), Aatomoxetine (25.21%), and aripiprazole (21.49%; Table 3).

**TABLE 3:** Preferred drugs during treatment (N = 242).

Drugs	TD patients without Comorbidities		TD patients with ADHD	
	Number(n)	Ratio (%)	Number(n)	Ratio (%)
Tiapride	147	60.74	123	50.83
Sulpiride	30	12.40	28	11.57
Haloperidol	62	25.62	61	25.21
Pimozide	5	2.07	6	2.48
Clonidine	79	32.64	79	32.64
Guanfacine	1	0.41	5	2.07
Aripiprazole	40	16.53	52	21.49
Risperidone	15	6.20	31	12.81
Ziprasidone	1	0.41	2	0.83
Olanzapine	6	2.48	7	2.89
Quetiapine	0	0.00	5	2.07
Topiramate	15	6.20	27	11.16
Sodium valproate	30	12.40	44	18.18
Levetiracetam	16	6.61	27	11.16
Aatomoxetine	-	-	61	25.21
Methylphenidate	-	-	36	14.88

When selecting therapeutic drugs, physicians cited the following factors as priority considerations: clinical guideline recommendations (4.55 points), better clinical efficacy (4.44 points), fewer adverse drug reactions (4.38 points), sufficient clinical evidence (4.24 points), convenient dosage forms (4.11 points), and better patient adherence (4.10 points; Table 4).

**TABLE 4:** Considerations of choosing in selecting drugs (N = 242).

Factors	1 n (%)	2 n (%)	3 n (%)	4 n (%)	5 n (%)	Average score
Clinical guideline recommendations	6 (2.48)	2 (0.83)	18 (7.44)	44 (18.18)	172 (71.07)	4.55
Recommended by experts	9 (3.72)	10 (4.13)	58 (23.97)	93 (38.43)	72 (29.75)	3.86
Sufficient clinical evidence	6 (2.48)	8 (3.31)	28 (11.57)	79 (32.64)	121 (50.00)	4.24
Better clinical efficacy	7 (2.89)	4 (1.65)	18 (7.44)	59 (24.38)	154 (63.64)	4.44
Fewer adverse drug reactions	8 (3.31)	5 (2.07)	19 (7.85)	65 (26.86)	145 (59.92)	4.38
Convenient use of dosage forms	9 (3.72)	10 (4.13)	38 (15.7)	73 (30.17)	112 (46.28)	4.11
Smaller drug dose	14 (5.79)	24 (9.92)	59 (24.38)	56 (23.14)	89 (36.78)	3.75
Better drug tastes	22 (9.09)	27 (11.16)	61 (25.21)	51 (21.07)	81 (33.47)	3.59
Better drug appearance	77 (31.82)	51 (21.07)	52 (21.49)	35 (14.46)	27 (11.16)	2.52
Cheaper drug price	19 (7.85)	35 (14.46)	89 (36.78)	49 (20.25)	50 (20.66)	3.31
More plentiful supplies in hospital	20 (8.26)	13 (5.37)	69 (28.51)	64 (26.45)	76 (31.40)	3.67
Patients’ demands	12 (4.96)	29 (11.98)	80 (33.06)	72 (29.75)	49 (20.25)	3.48
High degree of patient recognition	7 (2.89)	17 (7.02)	63 (26.03)	85 (35.12)	70 (28.93)	3.80
Better patient adherence	6 (2.48)	7 (2.89)	43 (17.77)	88 (36.36)	98 (40.50)	4.10

Note: 1 point means very unimportant, 5 points means very important.

### 3.2 Survey of Patient Guardians

#### 3.2.1 Patient Information

A total of 621 questionnaires were collected, of which 610 contained complete responses (effective rate: 98.2%). Three quarters (77.90%, 475/610) of patients with TD were male. Patient age ranged from 2.20 to 15.98 years (mean: 7.86 ± 2.38 years). The mean course of TD disease was 1.44 ± 1.48 years, and 26.10% (159/610) of the patients had comorbidities. Disease types were transient TD (322/610, 52.80%), chronic TD (27.20%, 166/610), other (11.80%, 72/610), and Tourette syndrome (8.20%, 50/610).

#### 3.2.2 Guardians’ Knowledge of TD

More than half of the guardians had learned about TD through medical staff (53.60%, 327/610) and self-education (52.30%, 319/610). Most guardians (81.50%) thought that TD was a neuropsychiatric disease, while 5.10% (31/610) thought that TD was not a disease. More than 80% of the guardians believed that the cause of TD was neurotransmitter imbalance. The factors that guardians thought would aggravate tic symptoms were stress (81.10%, 495/610), shock (72.30%, 441/610), being reminded (51.60%, 315/610), fatigue (49.30%, 301/610), concentration (20.20%, 123/610), infections (1.30%, 8/610), colds (0.80%, 5/610), and watching television or using electronic devices (0.80%, 5/610). As for common treatments for TD, most guardians were aware of drug therapy (89%, 543/610) and psycho-behavioral therapy (78.20%, 477/610), but fewer knew about educational interventions (29.30%, 179/610), physical therapy (19.50%, 119/610), or surgery (1.50%, 9/610). More than half of the guardians believed that TD treatment lasted 6–12 months (Table 5).

**TABLE 5:** Guardian’s cognition of TD (N = 610).

Option	Number(n)	Proportion (%)
Understanding TD Pathways (Multiple Choices)		
Medical staff’s information	327	53.60
Discovery by themselves	319	52.30
Other patients’ information	59	9.70
The Internet	16	2.60
Don’t understand the disease	11	1.80
Books	3	0.50
What type of disease is TD?		
Neuropsychiatric disease	497	81.50
Psychological disease	50	8.20
Not a disease	31	5.10
Otolaryngology disease	13	2.10
Ophthalmic disease	10	1.60
Unclear	5	0.80
Respiratory diseases	4	0.70
TD symptoms and Characteristics (multiple choices)		
The involuntary, sudden and rapid contraction movement of the head, face, trunk and limbs	498	81.60
The sound like burping or coughing through the nose, mouth and throat	354	58.00
TD mostly started in childhood	267	43.80
New forms of tic may appear	182	29.80
It can occur when the motor system functions normally	122	20.00
Causes of TD (multiple choices)		
Neurotransmitter imbalance	510	83.60
Infectious immune factor	155	25.40
Genetic factor	150	24.60
Organic factor	59	9.70
Stress	19	3.10
Psychological factors	10	1.60
Malnutrition	6	1.00
Factors aggravating tic symptoms (multiple choices)		
Stress	495	81.10
Shock	441	72.30
Being reminded	315	51.60
Fatigue	301	49.30
Focusing attention	123	20.20
Infections	8	1.30
Colds	5	0.80
Watching television or using electronic devices	5	0.80
TD common treatment (multiple choices)		
Drug therapy	543	89.00
Psychobehavioral therapy	477	78.20
Educational intervention	179	29.30
Physical therapy	119	19.50
Surgery	9	1.50
TD treatment time		
About 1 month	58	9.50
About half a year	193	31.60
About 1 year	176	28.90
About 3–5 years	156	25.60
About 10 years	17	2.80
About 20 years	1	0.20
Lifetime	9	1.50

#### 3.2.3 Medical Behavior and Drug Provision by Guardians

Only 14.10% (86/610) of patients had received medical treatment immediately after the first onset of tics, and more than half of patients first received medical treatment at a neurology department (62.10%, 379/610). Only 51.50% (314/610) of the guardians participated in medication choices: 38.90% (237/610) of the guardians had expressed their medication preferences to physicians, and 66.40% (405/610) of the guardians took their children’s medication preference into consideration. In terms of medication behavior, 67.40% (411/610) of guardians thought that medication should be taken on time and at a regular dose, 66.70% (407/610) of the guardians immediately consulted medical staff when they observed new symptoms, and 23.60% (144/610) of the guardians thought that drug use should be discontinued or reduced when symptoms were alleviated. Moreover, 9.80% (60/610) thought that medication was unnecessary because they could manage the disorder themselves, and 2.30% (14/610) thought that medication was only necessary at the onset of tics (Table 6).

**TABLE 6:** Guardian’s medical behavior and medication choices (N = 610).

Option	Number(n)	Ratio (%)
For TD, which Department did You Go to at the First Time?		
Neurology department	379	62.10
Ophthalmology department	93	15.20
Pediatric department	34	5.60
Developmental-behavioral pediatrics	30	4.90
Otolaryngological department	27	4.40
Psychiatry department	26	4.30
Pneumology department	15	2.50
Psychological counseling department	4	0.70
Traditional Chinese medicine department	1	0.20
Other	1	0.20
The time between the onset of TD and the time to seek medical treatment		
More than 1 year	112	18.40
Several months	238	39.00
A few weeks	174	28.50
Immediately	86	14.10
Involvement in medication choices		
Yes	314	51.50
No	213	34.90
Uncertain	83	13.60
Whether you expressed your personal medication preferences to your physicians		
yes	237	38.90
No	265	43.40
Uncertain	108	17.70
Do you consider the child’s medication preference		
Yes	405	66.40
No	100	16.40
Uncertain	105	17.20
Correct medication for TD (multiple choices)		
Take medicine on time and in regular dose	411	67.40
Consult your doctor or pharmacist immediately if any new symptoms occur during medication	407	66.70
drug use should be discontinued or reduced when symptoms improved were alleviated	144	23.60
medication was unnecessary because disease they could manage the disorder themselves	60	9.80
Medication only needs to be taken during an onset of tic	14	2.30

#### 3.2.4 Medical Preferences of Guardians

When selecting medications, guardians placed emphasis on drugs with fewer adverse reactions (4.52 points), recommendations from physicians (4.44 points), better clinical efficacy (4.29 points), lower drug doses (4.27 points), more convenient dosage forms (4.01 points), and sufficient supplies at the hospital (3.95 points; Table 7).

**TABLE 7:** Factors to consider in the medication choices of the patient’s guardian (N = 610).

Factor	1 n (%)	2 n (%)	3 n (%)	4 n (%)	5 n (%)	Average score
Recommended by physicians	4 (0.7)	5 (0.8)	32 (5.2)	244 (40.0)	325 (53.3)	4.44
Recommended by other patients	22 (3.6)	132 (21.6)	214 (35.1)	180 (29.5)	62 (10.2)	3.21
Better clinical efficacy	3 (0.5)	10 (1.6)	59 (9.7)	275 (45.1)	263 (43.1)	4.29
drugs with fewer adverse reactions	6 (1.0)	0 (0.0)	35 (5.7)	198 (32.5)	37 (60.8)	4.52
Convenient use of dosage forms	2 (0.3)	27 (4.4)	128 (21.0)	259 (42.5)	194 (31.8)	4.01
Smaller drug dose	3 (0.5)	10 (1.6)	66 (10.8)	271 (44.4)	260 (42.6)	4.27
Better drug tastes	9 (1.5)	99 (16.2)	249 (40.8)	168 (27.5)	85 (13.9)	3.36
Better drug appearance	53 (8.7)	264 (43.3)	216 (35.4)	53 (8.7)	24 (3.9)	2.56
Cheaper drug price	11 (1.8)	78 (12.8)	212 (34.8)	205 (33.6)	104 (17.0)	3.51
More plentiful supplies in hospital	3 (0.5)	33 (5.4)	119 (19.5)	299 (49.0)	156 (25.6)	3.95

Note: 1 point means very unimportant, 5 points mean very important.

## 4. Discussion

### 4.1 Main Findings

The majority of physicians we polled thought that the most important treatment goals for patients with TD were to improve their overall function, reduce the frequency of tics, and control comorbidities. The most important treatment strategies include the provision of effective strategies to manage TD, oral or written education of both patients and guardians, and medication. Psycho-behavioral therapy, educational interventions, and medication are the main treatment methods. The first-line drugs include selective D2 dopamine receptor antagonists (e.g., tiapride), α-adrenergic agonists (e.g., clonidine), and antipsychotics (haloperidol and aripiprazole). Clinical guidelines, better clinical efficacy, fewer adverse drug reactions, sufficient clinical evidence, convenient dosage forms, and better patient adherence are the important factors influencing the medication choices of pediatricians. Haloperidol was used for patients with severe tics, which was recommended as a second-line drug in Chinese guideline (Liu et al., 2020), and weak recommendations are made for the use of haloperidol in Canadian guideline (Pringsheim et al., 2012), doctors also prescribed trihexyphenidyl to reduce extrapyramidal reactions caused by haloperidol in China. Chinese pediatricians’ drug choices for treating TD generally follow clinical recommendations (Pringsheim et al., 2012; Pringsheim et al., 2019; Liu et al., 2020), but they do not fully consider guardian preferences and medication prices when selecting drugs. This may reflect the heavy workload of physicians and the short time allotted to each patient visit, precluding in-depth communications between physicians and patients or guardians, further improvements and optimizations are required in future medical practice. Tiapride is not a very common medicine in western countries, but it was recommended as a first-line drug for TD in Chinese guideline (Liu et al., 2020), adequate clinical research evidence showed that the drug is effective and safe, and it is also very cheap in China, so it is widely used.

We found that guardians had a poor understanding of the disease, especially its classifications, symptoms, and characteristics, factors aggravating tic symptoms, and the length of treatment. In addition, the guardians were not sufficiently aware of educational interventions, physical therapy, or surgical options, and some guardians even misunderstood treatment needs. Because of the guardians’ poor understanding of TD, some patients did not receive timely medical attention when needed, delaying treatment. Therefore, more effective education should be provided to patients and their guardians to enhance their cognition of TD. Moreover, some guardians did not understand the nature of the drug therapy, believing that medication could be discontinued or the dose reduced when symptoms were alleviated; some even believed that medication was only required at the onset of tic. In terms of medication choices, both guardians and clinicians preferred drugs with fewer adverse effects and better clinical efficacy, but guardians also considered factors such as smaller drug dose, more convenient dosage forms, and a steady and convenient supply. However, guardians reported that most physicians did not consider patients’ treatment needs, underscoring the importance of physicians listening to guardians’ input when making medication choices.

### 4.2 Comparisons With Other Studies

A Japanese survey from 2019 (Yu et al., 2019) found that the most important factor considered in the decision to begin pharmacotherapy in children with TD was functional impairment caused by tic symptoms, and this finding is consistent with ours. Aripiprazole and risperidone were the first- and second-line medications for TD, as α-adrenergic agonists are seldom prescribed in Japan, although they are widely used in China. This difference in clinical practice may result from the fast-acting receptor agonist clonidine being the only α-adrenergic receptor agonist officially accepted for treatment of hypertension in Japan. In addition, Aatomoxetine was a first-line drug because the use of methylphenidate is restricted in Japan.

A cross-sectional study of TD medications prescribed in Korea between 2009 and 2016 (Choi et al., 2019) reported that aripiprazole was the most commonly prescribed drug, the use of risperidone was declining, and the number of prescriptions written increased over time. Other commonly used drugs were benzatropine and haloperidol. The widespread use of aripiprazole might be related to the mounting body of evidence that indicates that aripiprazole has good efficacy and tolerability. In China, physicians’ drug choices are similar, with the exception of benzatropine and haloperidol, which are rarely used because of the high incidence of adverse drug reactions.

Lu et al. (2020) recently surveyed 110 pediatricians in China on drug treatment of patients with newly diagnosed TD and comorbidities. Their findings were consistent with ours, although their sample was smaller and they did not report factors that influenced medication choices. Geng et al. (2016) surveyed 57 guardians on their knowledge of TD; 71.90% believed that TD was a disease, but 73.70% still adopted inappropriate measures when tics occurred, indicating that guardians had a poor understanding of TD, similar to our findings. However, the study did not investigate patients’ medication needs or factors in medication choices.

### 4.3 Limitations of the Study

Our study has some limitations. First, we did not sample at random, but we did include physicians from 24 provinces and Hong Kong, Macao, and Taiwan. Guardians were recruited from the largest women and children’s hospital in southwestern China, so the results were of good representativeness. Second, we used a cross-sectional design to identify the factors influencing medication choices, so causal inference could not be made. Third, patient medications were reported by guardians. Although this can reflect patients’ medication needs to a certain extent, some information bias was inevitable. Fourth, this study is from a specific region, so the extrapolation has certain limitations. Future research should overcome these limitations.

### 4.4 Conclusion

We found that pediatricians in China typically follow clinical guidelines in selecting medications for TD but seldom consider guardian preferences, highlighting a gap in optimizing treatment. Moreover, patient guardians lack sufficient knowledge of TD and medication choices, requiring more physician-initiated dialogue.

## Data Availability

The original contributions presented in the study are included in the article/Supplementary Material, and further inquiries can be directed to the corresponding authors.

## Abbreviations

TD, tic disorder.
